# Single-molecule fluorescence in-situ hybridization reveals that human SHANK3 mRNA expression varies during development and in autism-associated SHANK3 heterozygosity

**DOI:** 10.1186/s13287-018-0957-3

**Published:** 2018-07-31

**Authors:** Samuel E. Taylor, Ruth D. Taylor, Jack Price, Laura C. Andreae

**Affiliations:** 1 Centre for Developmental Neurobiology, Institute of Psychiatry, Psychology and Neuroscience, King’s College London, London, SE1 1UL UK; 2 MRC Centre for Neurodevelopmental Disorders, King’s College London, London, UK; 3 Department of Basic and Clinical Neuroscience, Institute of Psychiatry, Psychology and Neuroscience, King’s College London, London, SE5 8AF UK

**Keywords:** Single-molecule fluorescence in-situ hybridization, mRNA, SHANK3, ProSAP2, Human inducible pluripotent stem cell, Autism, Autism spectrum disorder

## Abstract

**Background:**

Deletions and mutations in the *SHANK3* gene are strongly associated with autism spectrum disorder and underlie the autism-associated disorder Phelan–McDermid syndrome. SHANK3 is a scaffolding protein found at the post-synaptic membrane of excitatory neurons.

**Methods:**

Single-molecule fluorescence in-situ hybridization (smFISH) allows the visualization of single mRNA transcripts in vitro*.* Here we perform and quantify smFISH in human inducible pluripotent stem cell (hiPSC)-derived cortical neurons, targeting the *SHANK3* transcript.

**Results:**

Both smFISH and conventional immunofluorescence staining demonstrated a developmental increase in SHANK3 mRNA and protein, respectively, in control human cortical neurons. Analysis of single *SHANK3* mRNA molecules in neurons derived from an autistic individual heterozygous for *SHANK3* indicated that while the number of *SHANK3* mRNA transcripts remained comparable with control levels in the cell soma, there was a 50% reduction within neuronal processes, suggesting that local, dendritic targeting of *SHANK3* mRNA may be specifically affected in *SHANK3* haploinsufficiency.

**Conclusion:**

Human *SHANK3* mRNA shows developmentally regulated dendritic localization in hiPSC-derived neurons, which is reduced in neurons generated from a haploinsufficient individual with autism. Although further replication is needed, given the importance of local mRNA translation in synaptic function, this could represent an important early abnormality.

## Background

Haploinsufficiency for the *SHANK3/ProSAP2* gene is believed to be one of the most common monogenic causes of autism spectrum disorder (ASD), accounting for approximately 0.5% of cases [1]. Deletions or mutations in *SHANK3* underlie the autism-associated neurodevelopmental disorder Phelan–McDermid syndrome (PMS) [2, 3] and have also been strongly associated with non-syndromic ASD [4–6]. The SHANK proteins are synaptic scaffolding proteins that are enriched at the post-synaptic density of excitatory synapses, where they interact with other post-synaptic density proteins to influence synapse structure and function [7–9]. Studies in both mice and human cell lines where SHANK3 is deleted have revealed multiple deficits in excitatory synapse function, as well as intrinsic neuronal abnormalities [10, 11]. However, although there is evidence that overall levels of *SHANK3* mRNA are reduced in human inducible pluripotent stem cell (hiPSC)-derived neurons from patients with heterozygous *SHANK3* deletions and PMS [12], little is known regarding the local expression of *SHANK3* mRNA in human neurons. While *shank3* mRNA has been detected in the neuropil of hippocampal CA1 pyramidal neurons in rodents, presumably corresponding to dendrites [13, 14], no detailed analysis has been done in humans. We therefore utilized a single-molecule fluorescent in-situ hybridization (smFISH) approach in neurons derived from hiPSCs to examine *SHANK3* expression in more detail.

Single-molecule fluorescent in-situ hybridization (smFISH) uses a combination of multiple, small, fluorescently labelled probes, each probe complementary to a different region along the nucleic acid of interest, to increase detection sensitivity and allow the visualization of single nucleic acid molecules. This technique has been used to detect single RNA molecules in a wide range of cells and organisms, from yeast [15] to mouse intestinal stem cells [16], and more recently in humans to detect expanded repeats in polyglutamine diseases [17] and long non-coding RNAs in fibroblasts and HeLa cells [18]. Here, we designed a combination of 48 unique smFISH probes to detect human *SHANK3* mRNA transcripts. We used hiPSC-derived neurons from a control iPSC line [19] which were differentiated to a cortical fate using a well-validated protocol [20]. We quantified both SHANK3 mRNA and protein levels in the cell soma and in neuronal processes at different developmental time points as the neurons mature in culture. Finally, to investigate whether there are compartment specific reductions in *SHANK3* mRNA in the context of SHANK3 haploinsufficiency, we examined the localization of single *SHANK3* mRNA molecules in neurons derived from an individual with autism (but not PMS) with a microdeletion affecting only the *SHANK3* gene [19, 21].

## Methods

### Cell culture

Human inducible pluripotent stem cell (hiPSC) lines were generated from keratinocytes using a lentiviral construct [19]. Neural induction to produce cortical neuronal progenitors was performed using a modified dual SMAD inhibition protocol [20, 21]. *SHANK3*^+/−^ hiPSCs were generated from a 4-year-old male with a deletion on chromosome 22q extending from the third intron of *SHANK3* through to past the 3′ end of the gene [19]. All time points mentioned begin from the point of final plating as neuronal progenitors. All consumables were purchased from Gibco unless otherwise stated.

Prior to plating, Grid-500 plates (ibidi GmbH) were coated with poly-d-lysine 70–150 kDa (PDL, 5 μg/ml; Sigma) incubated at 37 °C for 6 h, followed by three washes with PBS and laminin coated overnight (10 μg/ml) at 37 °C. Neuronal cultures were thawed in rho kinase (ROCK) inhibitor (10 μM) and *N*-((3,5-difluorophenyl)acetyl)-l-alanyl-2-phenylglycine-1,1-dimethylethyl ester (DAPT, 10 μM) in hiPSC media (2 mM glutamax, 50 mg/ml penicillin, 50 mg/ml streptomycin, in Neurobasal medium supplemented with B27 following the manufacturer’s instructions), centrifuged at 160 × *g* for 5 min and the supernatant re-suspended in ROCK inhibitor media. Human cortical neuronal progenitors were plated at 312,000 cells/cm^2^ and incubated at 37 °C. At 1 and 4 days in vitro (DIV), half of the media was replaced with DAPT (10 μM) in hiPSC media. At 7 DIV, half of the media was replaced with brain-derived neurotrophic factor (BDNF, 10 ng/ml; PeproTech) in hiPSC media and rat cortical glia coverslips were placed inverted onto the neuronal cultures. Thereafter, half of the culture media was replaced with fresh BDNF media twice a week.

To produce rat cortical glia coverslips, rat cortical glial cells were generated according to the method described by Kaech and Banker [22]. Embryonic day 18 Sprague Dawley rats were obtained from pregnant dams purchased from Charles River. Briefly, rat cortices were triturated in trypsin–EDTA and grown in DMEM-based glial media (2.4 mg/ml glucose, 50 mg/ml penicillin, 50 mg/ml streptomycin, 10% heat-inactivated horse serum) and plated on pre-coated 30–70 kDa PDL (5 μg/ml) T75 flasks at 100,000 cells/cm^2^. Rat glia were then incubated for 2 weeks at 37 °C with growth media replaced twice a week. Glia were then passaged with trypsin–EDTA for 5 min at 37 °C, and plated on to 70–150 kDa PDL-coated (5 μg/ml) glass coverslips with paraffin wax pedestals at 100,000 cells/cm^2^. Glial coverslips were then incubated for 1 week (37 °C) before co-culturing with hiPSC-derived neuron plates.

### Immunofluorescence

Immunofluorescence was carried out as described previously [23]. All consumables were purchased from Gibco unless otherwise stated. Briefly, cells were fixed in 4% paraformaldehyde with 10 mg/ml sucrose for 15 min, permeabilized in 0.01% saponin (Sigma) for 5 min, blocked in 3% bovine serum albumin (BSA-block; Sigma) for 30 min and incubated overnight in primary antibodies diluted in blocking solution at 4 °C.

The primary antibodies used were rabbit-anti-SHANK3 (1:500; Atlas Ab), mouse IgG1-anti-GFAP (1:500; Millipore) and mouse IgG1-anti-MAP2 (1:500; Sigma). Cells were then washed in PBS before incubation in secondary antibodies for 30 min at room temperature. The secondary antibodies used were donkey-anti-mouse Alexa Fluor 568 and 647 (1:1000) and donkey-anti-rabbit Alexa Fluor 488 (1:1000). Nuclear DAPI staining was also carried out. Images were acquired with an Olympus FV1000 confocal microscope using the Fluoview software and a 60× oil immersion objective (1.4 numerical aperture).

### Single-molecule fluorescence in-situ hybridization

Single-molecule fluorescence in-situ hybridization (smFISH) was carried out according to the Stellaris RNA smFISH protocol (Bioscience Technologies). All solutions used were made up using nuclease-free water and consumables purchased from Ambion unless otherwise stated. Growth medium was initially aspirated from the neuronal culture dishes and the cells washed in PBS. Cells were then fixed in 4% formaldehyde for 10 min at room temperature and washed twice in PBS, followed by 1-h incubation at 37 °C in 70% ethanol. Wash buffer (10% deionized formamide) in 2× saline-sodium citrate (SSC) was then added for 2–5 min at room temperature followed by 1:100 *SHANK3* mRNA smFISH probe in hybridization buffer (10% deionized formamide, 0.1 g/ml dextran sulfate; Millipore) in 2× SSC and incubated in the dark within a humidity chamber for 16 h at 37 °C. The custom *SHANK3* smFISH mRNA probe (ST_SHANK3, Rprobes w/Quasar 570, Custom Stellaris smFISH; Biosearch Technologies) consisted of 48 fluorescently labelled probes complementary to different regions of the *SHANK3* mRNA transcript. Each probe was 20 nucleotides in length. Cells were then incubated in wash buffer for 30 min at room temperature, washed twice in 2× SSC and stored in 2× SSC at 4 °C. Images were acquired as already described.

### SHANK3 mRNA and protein quantification

**Fig. 1:**
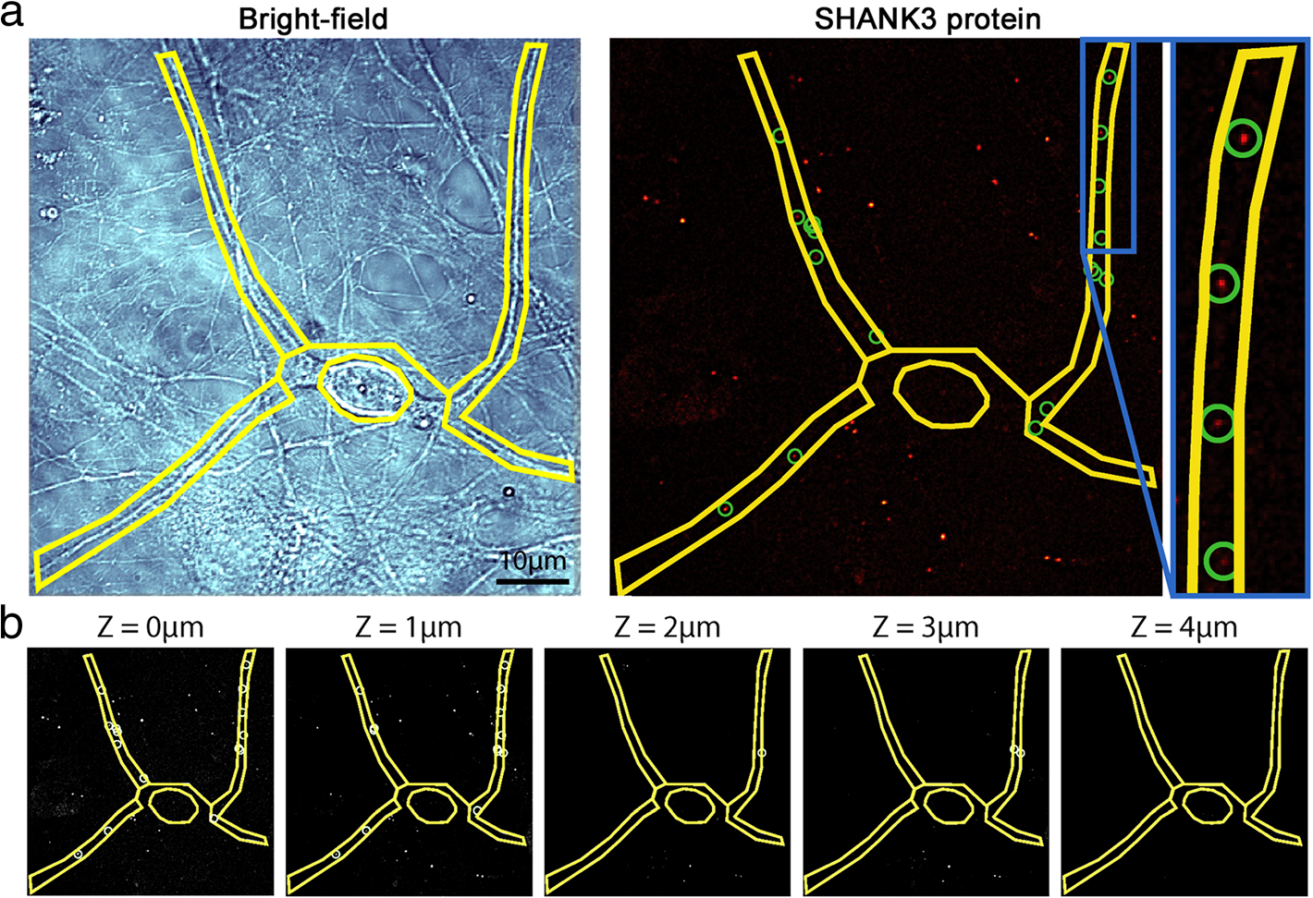
SHANK3 mRNA and protein puncta selection in hiPSC-derived cortical neurons. **a** Left panel: bright-field images used to manually outline cell body and neuronal processes. Right panel: puncta (circled) detected using automated algorithm, defined by intensity and size threshold. Only puncta lying within outlined areas included. **b** Puncta detected at each plane (1-μm intervals) per *Z*-stack. SHANK3 SH3 and multiple ankyrin repeat domains 3

The number of *SHANK3* mRNA and protein puncta was quantified within the cell body and processes of hiPSC-derived neurons using the FISHquant software custom MATLAB script (FISH-quant v2c, MATLAB package [24]). Bright-field images were used to outline both the neuronal cell body and processes, and their respective areas calculated (Fig. 1). Bright-field images were used instead of immunofluorescence images as smFISH could not be performed in combination with immunofluorescence without diminishing the smFISH signal. Puncta within the outlined areas of the bright-field images were counted at each layer of the *Z*-stack and the total number of puncta summed. A set of parameters including puncta size, pixel intensity and distance between puncta was used to minimize the selection of false-positive puncta. A region of background was analysed as a further control to the puncta selection parameters. Average puncta density was then calculated for each region.

## Results

### SHANK3 protein and mRNA expression within hiPSC-derived neurons

**Fig. 2:**
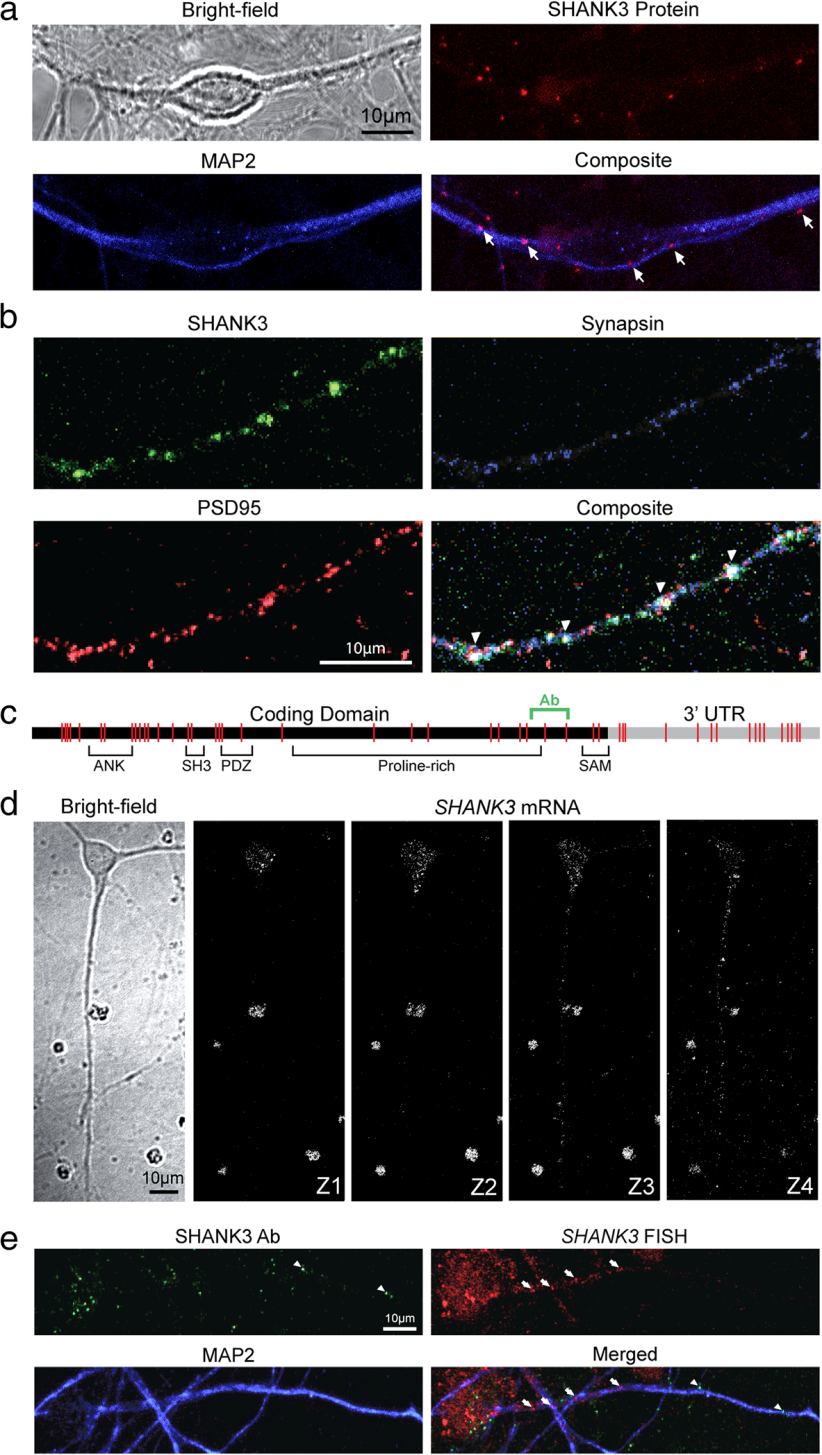
SHANK3 mRNA and protein labelling and co-localization in hiPSC-derived neurons. **a** Immunofluorescence labelling of SHANK3 protein (red) in MAP2-positive dendrites (blue) of hiPSC-derived neurons. Arrows mark co-localization of SHANK3 protein. **b** SHANK3 (green) co-localizes with the pre-synaptic marker synapsin (blue) and post-synaptic marker PSD95 (red). Arrowheads mark co-localization of SHANK3 protein. **c** Schematic illustrating SHANK3 mRNA. Vertical red lines indicate complementary binding sites for smFISH probes along both coding domain and 3′-UTR. SHANK3 antibody binding site marked green. **d** Representative *Z*-stack of single *SHANK3* mRNA transcripts labelled by smFISH in 51 DIV hiPSC-derived neurons. *SHANK3* mRNA localized to cell body and processes in hiPSC-derived neurons. **e** Combined SHANK3 mRNA and protein labelling in 49-DIV hiPSC-derived neuron. White arrowheads label SHANK3 protein puncta. White arrows label *SHANK3* mRNA puncta. Ab antibody, FISH fluorescence in-situ hybridization, MAP2 microtubule-associated protein 2, SHANK3 SH3 and multiple ankyrin repeat domains 3, UTR untranslated region

Firstly, we used immunofluorescence staining to confirm SHANK3 protein expression in hiPSC-derived cortical neurons. Co-labelling with the dendritic marker MAP2 indicated SHANK3 expression localized to dendrites and was not present at the cell body (Fig. 2a). SHANK3 expression also co-localized with the excitatory postsynaptic marker PSD95, confirming that the SHANK3 labelling was specific to excitatory synapses (Fig. 2b). We then went on to examine *SHANK3* mRNA expression in hiPSC-derived neurons using single-molecule fluorescent in-situ hybridization (smFISH). A total of 48 smFISH unique probes were designed to bind to complementary sites located within both the *SHANK3* mRNA coding region and 3′-untranslated region (3’-UTR) (Fig. 2c). Confocal *Z*-stack images were taken 0.5 μm apart to retain high resolution with clear identification of individual puncta at each slice. *SHANK3* mRNA puncta were clearly visible in both cell soma and neuronal processes neurons throughout the *Z*-stack (Fig. 2d), although *SHANK3* mRNA and protein puncta did not co-localize (Fig. 2e).

### Developmental regulation of SHANK3 protein and mRNA expression

**Fig. 3:**
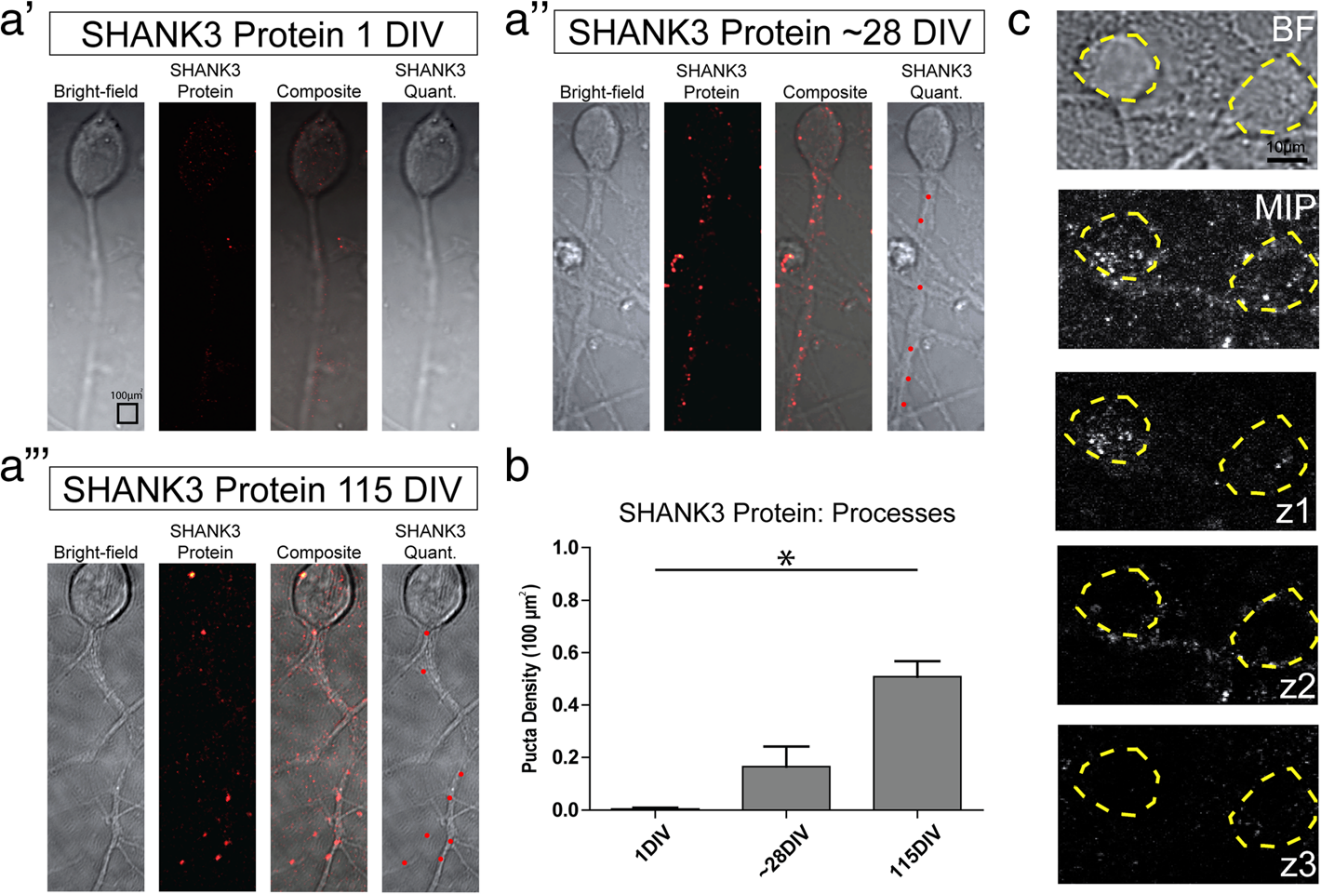
SHANK3 protein expression increases with developmental age. **a** SHANK3 protein identified through immunofluorescence labelling. For each panel, left to right: bright-field image (BF) of representative hiPSC-derived neuron, SHANK3 protein immunofluorescence image, composite immunofluorescence and BF image, and SHANK3 protein puncta selected through automated quantification analysis. Images for (a′) 1 day in vitro (DIV) (*n* = 3), (a′′) 28 DIV (*n* = 4) and (a′′′) 115 DIV (*n* = 5). **b** SHANK3 protein puncta per 100 μm^2^. Error bars indicate standard error of mean. *Kruskal–Wallis test *p* < 0.005 at 1 DIV vs 115 DIV. **c** *Z*-stack demonstrates SHANK3 protein only present on outside and not within cell body of hiSPC-derived neurons as seen in maximum image projection (MIP). *Z*-stack images taken at 3.0 μm spacing. Yellow dashed lines surround neuronal cell body. SHANK3 SH3 and multiple ankyrin repeat domains 3

**Fig. 4:**
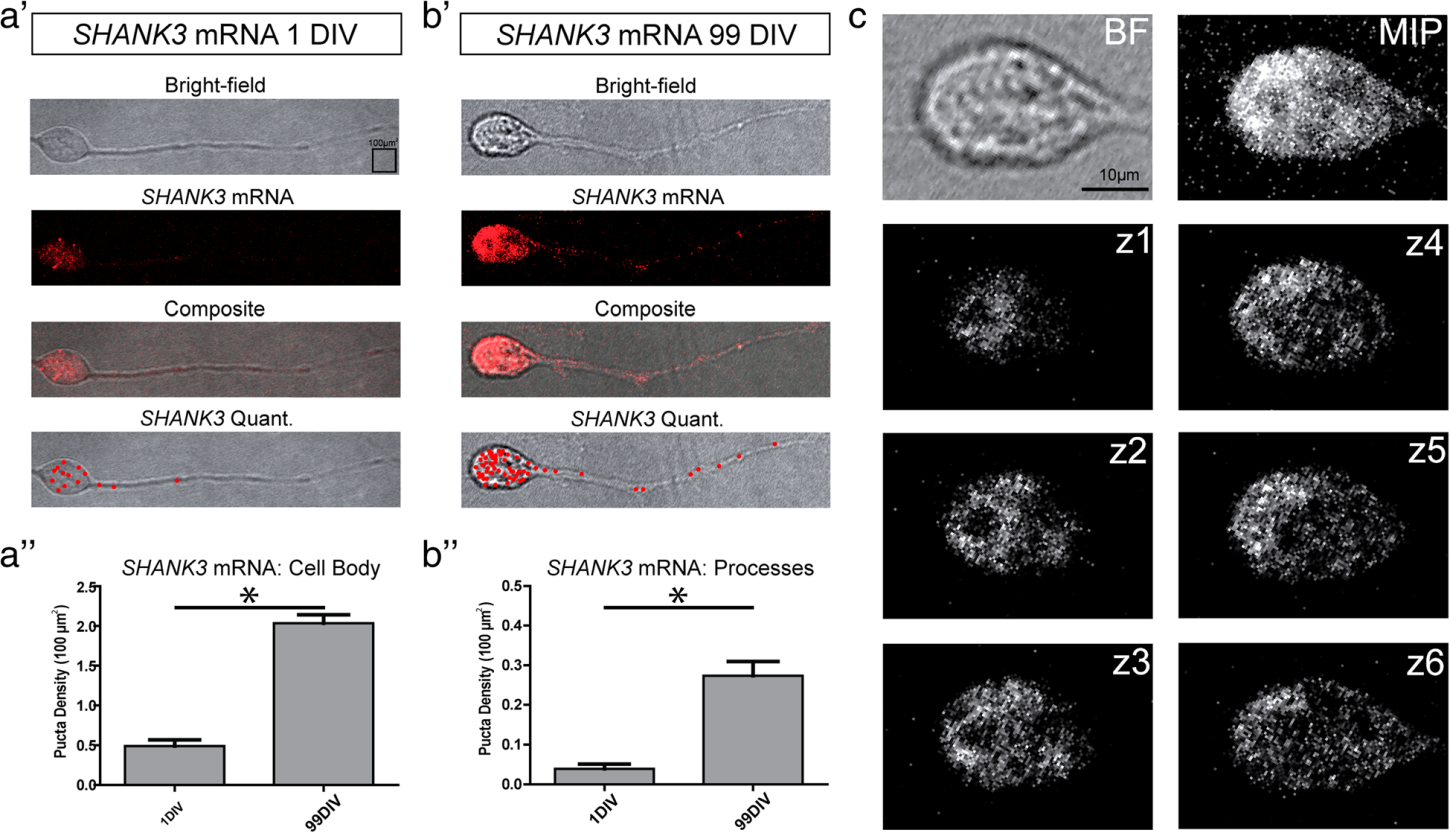
*SHANK3* mRNA puncta analysis in control hiPSC neurons over developmental time line. **a, b** *SHANK3* mRNA identified through smFISH. Top to bottom; bright-field (BF) image of representative hiPSC-derived neuron, *SHANK3* mRNA smFISH image, composite smFISH and BF image, and *SHANK3* mRNA puncta selected through quantification analysis. Images for (a′) 1 day in vitro (DIV) (left; *n* = 17) and (b′) 99 DIV (right; *n* = 11). Bar graph represents *SHANK3* mRNA puncta per 100 μm^2^ for (a”) cell body (left) and (b″) processes (right). Error bars indicate standard error of mean. *Unpaired *t*-test *p* < 0.0001. **c** *Z*-stack demonstrates *SHANK3* mRNA located throughout cell body of hiSPC-derived neurons as seen in maximum image projection (MIP). *Z*-stack images taken 1.0 μm apart. mRNA messenger RNA, SHANK3 SH3 and multiple ankyrin repeat domains 3

In order to determine the in-vitro timescale for synapse formation and neuronal maturation in our hiPSC-derived neurons, we carried out SHANK3 protein immunofluorescence staining at 1 DIV, 28 DIV, and 115 DIV (Fig. 3a, b). SHANK3 protein expression was not observed within neuronal cell bodies (Fig. 3c) and therefore SHANK3 protein puncta were only quantified within neuronal processes. We found that SHANK3 protein expression within neuronal processes significantly increased with age. The mean SHANK3 protein puncta density progressively increased from 0.0046 puncta/100 μm^2^ at 1 DIV (*n* = 3) to 0.1649 puncta/100 μm^2^ at 28 DIV (*n* = 4) and 0.5088 puncta/100 μm^2^ at 115 DIV (*n* = 5) (*p* < 0.005 at 1 DIV vs 115 DIV, Kruskal–Wallis test with Dunn’s multiple-comparison post test). Similarly, when we examined *SHANK3* mRNA expression with smFISH (at 1 DIV and 99 DIV), we found that this increased with neuronal age in both the cell body and in processes (Fig. 4a–c). For neuronal cell bodies, the mean *SHANK3* mRNA puncta density significantly increased from 0.5039 puncta/100 μm^2^ at 1 DIV (*n* = 17) to 2.412 puncta/100 μm^2^ at 99 DIV (*n* = 11) (*p* < 0.0001). For neuronal processes, the mean *SHANK3* mRNA puncta density significantly increased from 0.0392 puncta/100 μm^2^ at 1 DIV (n = 17) to 0.2737 puncta/100 μm^2^ at 99 DIV (n = 11) (*p* < 0.0001, unpaired *t* test). These results confirmed our expectations that both SHANK3 mRNA and protein would increase with neuronal maturation as synapses are progressively formed.

### Reduction in *SHANK3* mRNA expression in neurons derived from an individual with ASD and *SHANK3* haploinsufficiency restricted to neuronal processes

**Fig. 5:**
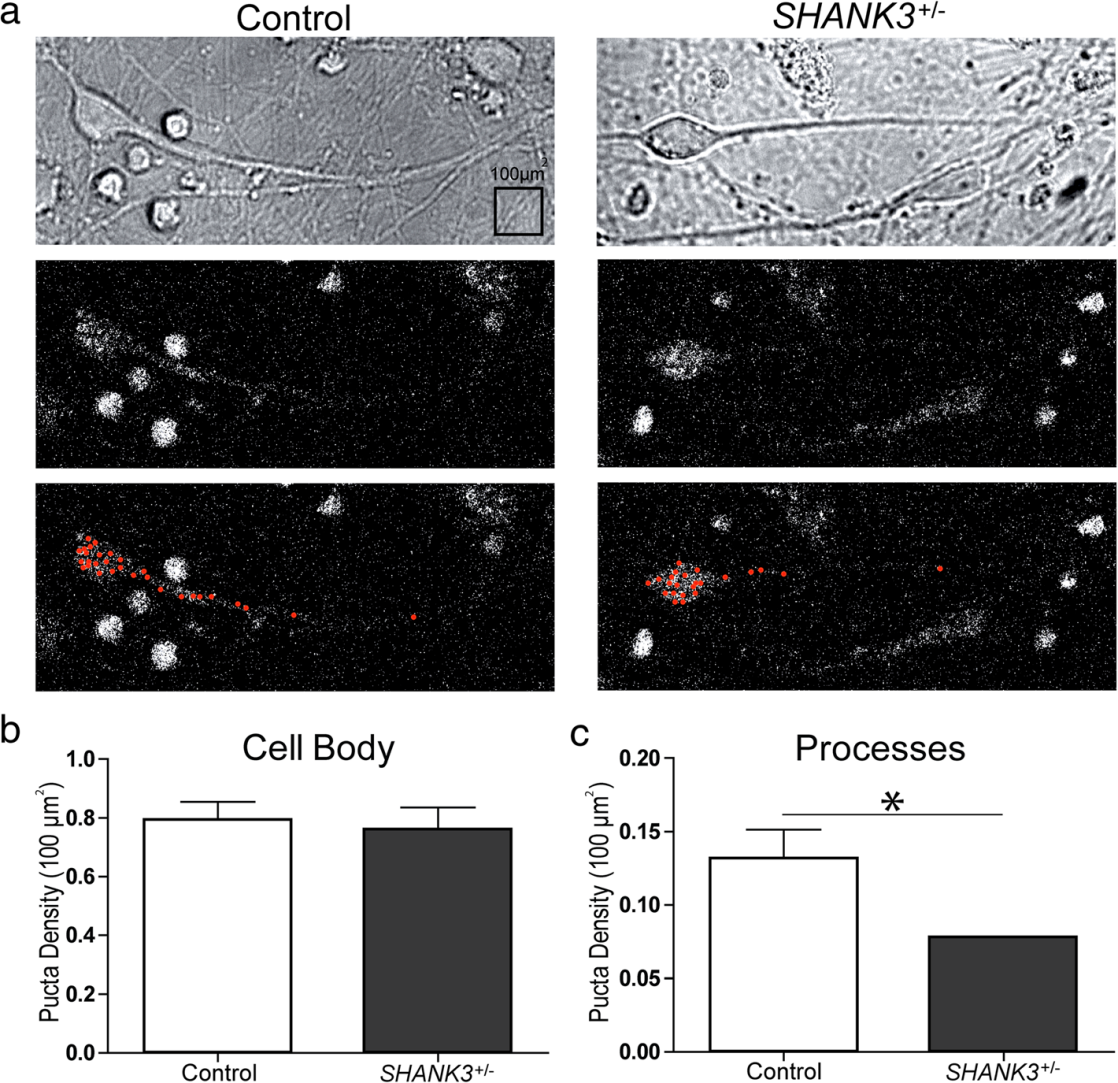
*SHANK3* mRNA expression in *SHANK3*^+/−^ male with ASD shows reduction restricted to processes. **a** Top to bottom: bright-field image, smFISH maximal projections (MIP) and MIP with identified puncta superimposed; both control (left) and *SHANK3*^+/−^ heterozygous patient (right) hiPSC-derived cortical neurons, 53 DIV. **b** *SHANK3* mRNA puncta per 100 μm^2^ within cell body of control (*n* = 16) and *SHANK3*^+/−^ heterozygous patient (*n* = 20) hiPSC-derived cortical neurons. **c** *SHANK3* mRNA puncta per 100 μm^2^ within processes of control (*n* = 16) and *SHANK3*^+/−^ heterozygous patient (*n* = 20) hiPSC-derived cortical neurons. *p = 0.0104 (unpaired *t* test). Errors bars indicate standard error of mean. SHANK3 SH3 and multiple ankyrin repeat domains 3

We then asked whether the number or localization of *SHANK3* mRNA molecules might be affected in the context of *SHANK3* haploinsufficiency. An overall reduction in *SHANK3* mRNA levels has been described previously in iPSC-derived neurons from patients with PMS using qRT-PCR [12], but their specific localization was not addressed. We used an hiPSC line generated from a 4-year-old male, diagnosed with ASD and developmental delay but not PMS, with a *SHANK3* gene deletion starting at the third intron and continuing just beyond the end of the gene on chromosome 22q [19]. We differentiated cortical neurons from the patient line and a control male hiPSC line, and examined *SHANK3* mRNA numbers and location at 53 DIV using smFISH. *SHANK3*^+/−^ patient hiPSC neurons exhibited almost a 50% reduction in *SHANK3* mRNA density within neuronal processes when compared with control (*SHANK3*^+/−^ patient mean mRNA density = 0.077 puncta/100 μm^2^ (*n* = 20), control mean = 0.132 puncta/100 μm^2^ (*n* = 16); *p* = 0.0104, unpaired *t* test) (Fig. 5a), while no differences were observed at the cell body (*SHANK3*^+/−^ patient mean = 0.794 puncta/100 μm^2^ (*n* = 20), control mean = 0.7624 puncta/100 μm^2^ (*n* = 16)) (Fig. 5b).

## Discussion

The idea that mRNA may be translated locally within dendrites of neurons grew from the visualization of synapse-associated polyribosome complexes (SPRCs) [25]. In-situ hybridization studies subsequently demonstrated the presence of specific mRNAs in neuronal dendrites [26] or dendrite-rich brain regions [27]. This profoundly changed the then dominant view that both transcription and translation occurred within the cell body [28]. Recent studies have shown upwards of over 2500 different mRNA transcripts locally translated within dendrites and axons of rat hippocampal neurons [13]. RNA binding proteins (RBPs) are thought to regulate the localization and translation of mRNA within dendrites by binding to the untranslated (3′-UTR and / or 5′-UTR) or coding regions [29]. Local translation in neuronal dendrites is believed to be important for synaptic modification, long-term potentiation (LTP) and long-term depression (LTD) [25, 30, 31]. Here we performed single-molecule fluorescence in-situ hybridization (smFISH) to label mRNA coding for the autism-risk gene *SHANK3*, and establish a protocol for puncta quantification. smFISH provides a higher sensitivity in molecular analysis and gene expression compared to traditional techniques, a level of sensitivity that has been lacking in hiPSC-derived lineages.

SHANK3 protein expression was quantified within our hiPSC-derived neurons to outline a developmental time line for pre-synapse and post-synapse formation. An increase in *SHANK3* mRNA in the neuronal processes was consistent with synapse formation and appears to be present in the processes before the formation of excitatory synapses. This direct visualization of individual SHANK3 mRNA confirms in human neurons previous findings from rodent models using conventional ISH [32] and high-resolution FISH [13], and supports the view that key synaptic genes such as SHANK3 are likely to undergo local translation in neuronal dendrites, similar to that seen in rodent models [13, 32].

Here we demonstrated almost a 50% decrease in *SHANK3* mRNA in the processes of SHANK3^+/−^ hiPSC neurons. SHANK3^+/−^ hiPSC neurons were generated from a male patient with a deletion on chromosome 22q which begins at the third intron of SHANK3 and extends beyond the end of the gene. As our data are from only one individual, it will be important to identify whether similar changes are seen in other SHANK3 deletion patients. In addition, it would be interesting to compare *SHANK3* haploinsufficiency in the absence of features of PMS, as studied here, with neurons from PMS patients. An overall reduction in *SHANK3* mRNA levels has been described previously in iPSC-derived neurons from patients with PMS using qRT-PCR [12], but their specific localization within the neurons was not addressed. The reduction in mRNA numbers in dendrites may have implications for the local translation of SHANK3. This finding is relevant as local dendritic translation of neuronal and synaptic proteins is believed to be important for synapse formation and function [25, 30, 31], both of which are often disrupted in disorders such as ASD [8, 33]. Indeed, abnormalities of dendritic mRNA translation in the ASD-associated disorder Fragile X syndrome are thought to result in changes to synapse function and plasticity [34–36]. *SHANK3* heterozygosity in human neurons has been shown to have multiple effects, including on synaptic number and function, neuronal morphology and intrinsic neuronal properties [11, 12, 21]. It would therefore be interesting in the future to examine whether, and to what extent, other mRNAs may exhibit differential localization.

## Conclusions

The ability to perform smFISH in hiPSC-derived neurons to label single target mRNA molecules provides a powerful way to study gene expression at high resolution in human cells. Our results confirm that previous findings, indicating the highly penetrant ASD-associated gene *SHANK3* is likely to undergo local dendritic translation in neurons, also hold true in human neurons. Further, we provide evidence that SHANK3 haploinsufficiency in human neurons may specifically affect mRNAs targeted to dendrites, although further studies will be needed to confirm to what extent this is seen in neurons from other individuals. Given the importance of local mRNA translation in synaptic function, this could represent a key early abnormality which merits further investigation.

## Abbreviations

ASD: Autism spectrum disorder
BDNF: Brain-derived neurotrophic factor
BSA: Bovine serum albumin
DAPI: 4′,6-Diamidino-2-phenylindole, dihydrochloride
DAPT: *N*-((3,5-Difluorophenyl)acetyl)-l-alanyl-2-phenylglycine-1,1-dimethylethyl ester
DIV: Days in vitro
DMEM: Dulbecco’s modified Eagle’s medium
hiPSC: Human inducible pluripotent stem cell
LTD: Long-term depression
LTP: Long-term potentiation
MAP2: Microtubule-associated protein 2
MIP: Maximum image projection
mRNA: Messenger RNA
PBS: Phosphate-buffered saline
PDL: Poly-d-lysine
PMS: Phelan–McDermid syndrome
ProSAP2: Proline-rich synapse-associated protein 2
qRT-PCR: Quantitative real-time polymerase chain reaction
RBP: RNA binding protein
ROCK: Rho kinase
SHANK3: SH3 and multiple ankyrin repeat domains 3
smFISH: Single-molecule fluorescence in-situ hybridization
SPRC: Synapse-associated polyribosome complex
SSC: Saline-sodium citrate
UTR: Untranslated region
